# Supplementary material for: Participation of the SMAD2/3 signalling pathway in the down regulation of megalin/LRP2 by transforming growth factor beta (TGF-ß1)
Source: PLoS One. 2019 May 23;14(5):e0213127. doi: 10.1371/journal.pone.0213127 (PMC6532859; doi:10.1371/journal.pone.0213127)
Supplement: S1 Fig — (PDF) [file pone.0213127.s001.pdf]

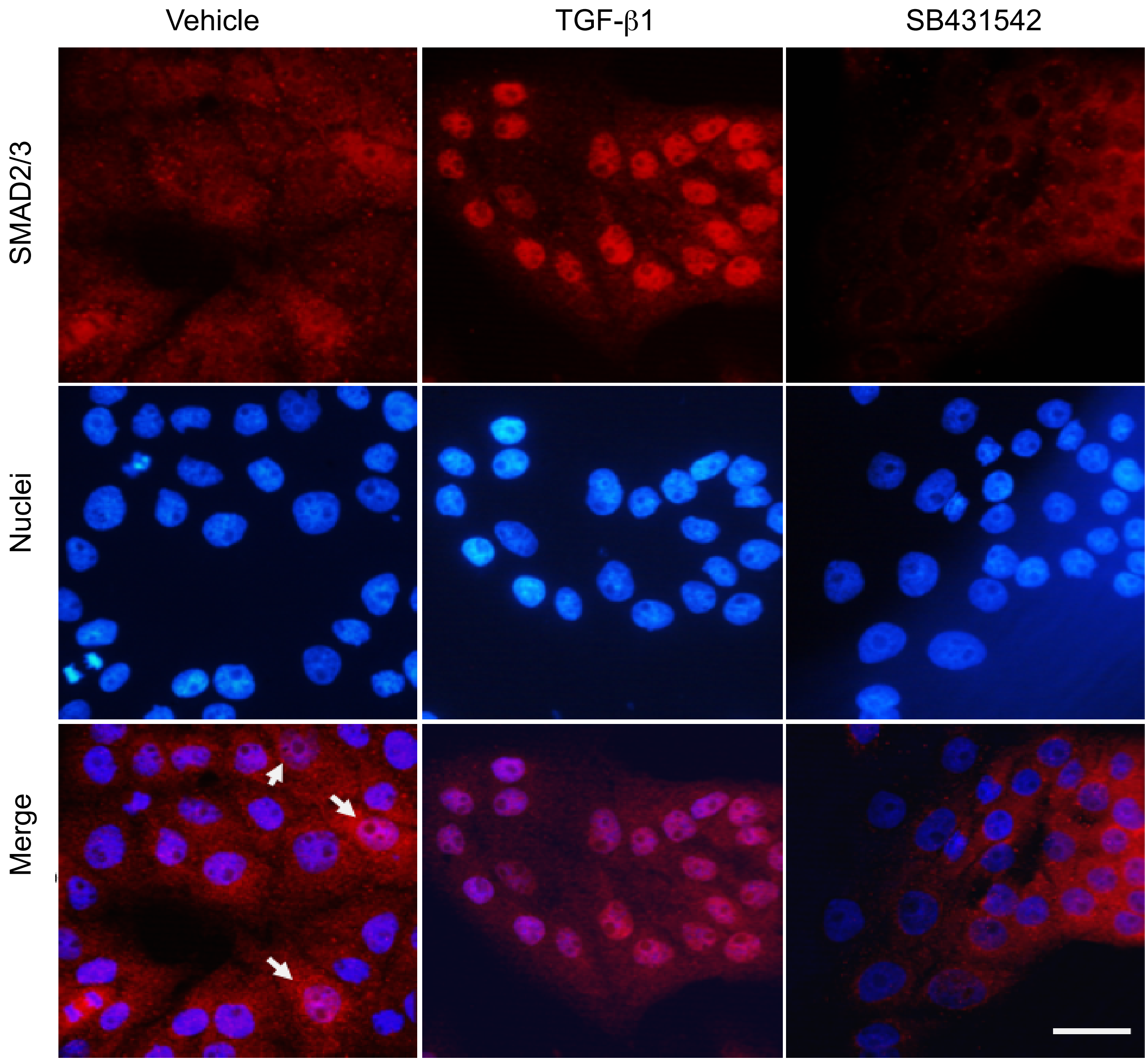

Fig S1: Subcellular distribution of SMAD2/3 in LLC-PK1 cells. LLC-PK1 cells were grown up to 70% confluence and were subsequently pre-treated with low serum (0.5% SBF) medium overnight followed by treatment with vehicle, TGF-β1 (2.5 ng/ml) or the TGF-βRI inhibitor SB-431542 (10 μM) for 6 hours. The expression and localization of SMAD2/3 (in red) was visualized with an antibody that detects total SMAD2/3. The images were obtained with a Leica DM2000 epifluorescence microscope. The arrows indicate the cells expressing SMAD2/3 in the nucleus under basal conditions. Scale bar 20 μm.
